# CFTR expression decreases with age in several airway cell types

**DOI:** 10.1038/s41598-024-80108-8

**Published:** 2024-11-21

**Authors:** Timothy E. Corcoran, Matthew J. Broerman, Corrine R. Kliment, Cecilia Lo

**Affiliations:** 1 Division of Pulmonary, Allergy, Critical Care, and Sleep Medicine, Department of Medicine, University of Pittsburgh, UPMC MUH NW628, 3459 Fifth Ave, Pittsburgh, PA 15213 USA; 2 Department of Bioengineering, University of Pittsburgh, Pittsburgh, PA USA; 3 Department of Chemical and Petroleum Engineering, University of Pittsburgh, Pittsburgh, PA USA; 4 Department of Pediatrics, University of Pittsburgh, Pittsburgh, PA USA

**Keywords:** CFTR, Mucus, Mucociliary clearance, Lung aging, Infection, Respiration

## Abstract

The mucociliary clearance (MC) system is a vital host defense against infection in the lung. MC system function is dependent on ciliary density, structure, and function and airway surface liquid (ASL) composition and hydration. Animal and human studies indicate that MC rate decreases with age which may contribute to the increased rates of pulmonary infection experienced by older people. The Cystic Fibrosis Transmembrane Conductance Regulator (*CFTR*) gene encodes an anion channel on epithelial surfaces that plays a key role in maintaining ASL hydration. Failure or dysfunction of CFTR could result in the dehydration of airway mucus, depressing MC. Here we use two available databases including bulk (GTEx) and single-cell (CELLxGENE) sequencing data from the lung to determine if *CFTR* expression decreases with age. Bulk expression data and single-cell expression data from goblet, club, and respiratory basal cells all demonstrated patterns of decreasing *CFTR* expression with age. Ciliated airway cells did not. Secretory cells (including club and goblet cells) and basal cells are the largest source of *CFTR* expression in the airway. This indicates that changes in *CFTR* expression and ASL dehydration may contribute to the decreasing MC associated with aging.

**Supplementary Information:**

The online version contains supplementary material available at 10.1038/s41598-024-80108-8.

## Introduction

The mucociliary clearance (MC) system is a vital host defense against obstruction and infection in the lung. The airways are lined with a protective layer of mucus that traps inhaled microbials and toxins. Airway cilia beat synchronously within a thin periciliary liquid layer underneath the mucus. The *metachronal wave* generated by the cilia pushes the mucus layer towards the central airways, clearing it from the lungs. The mucus and periciliary layers together comprise the airway surface liquid or ASL. MC system function is dependent on ciliary density, structure, and function^1^ and ASL composition^2^ and hydration^3^.

The Cystic Fibrosis Transmembrane Conductance Regulator (*CFTR*) gene encodes an anion channel on epithelial surfaces that plays a key role in maintaining ASL hydration. Secretion of chloride (Cl-) from CFTR promotes osmotic gradients that draw liquid from epithelial cells into the ASL. Cystic Fibrosis (CF) is associated with the absence or dysfunction of the CFTR protein which results in decreased epithelial Cl- secretion and ASL dehydration. The increased mucus concentrations in the dehydrated ASL can affect ciliary function, leading to MC depression or failure. This in turn leads to mucus accumulation and obstruction and an increased proclivity to opportunistic airway infections^3^. Decreases in CFTR function have also been associated with cigarette smoke and may play a role in COPD^4,5^.

Animal and human studies indicate that MC rate decreases with age^6–11^. This may contribute to the increased rates and severity of pulmonary infection experienced by older people^12–14^. Several different causes of age-related declines in MC have been proposed including decreases in ciliary beat frequency associated with oxidative stress, alteration of mucin composition, and changes in Cl- secretion^7,15,16^. Murine studies have demonstrated a decrease in ciliated cells with age and an increase in the ratio of club to ciliated cells^17^.

Here we explore whether *CFTR* expression changes with age in the lung using bulk RNA sequencing data from the adult Genotype Tissue Expression (GTEx) portal^18^ and single cell RNA sequencing data from the CELLxGENE single-cell human cell atlas which incorporates data from multiple single-cell databases including the Human Lung Cell Atlas (HLCA)^19^.

Decreasing *CFTR* expression may contribute to aging-related MC depression through ASL dehydration, like it does in CF lung disease. If this is the case, aging related MC depression might be restorable using a CFTR potentiator drug, such as ivacaftor, which was developed to treat CF^20^. Ivacaftor increases the open potential of the CFTR channel, allowing for more Cl- secretion from the same amount of CFTR. A first step in determining the potential of this approach is to understand how the expression of *CFTR* changes with age in the human airway.

## Results

**Fig. 1:**
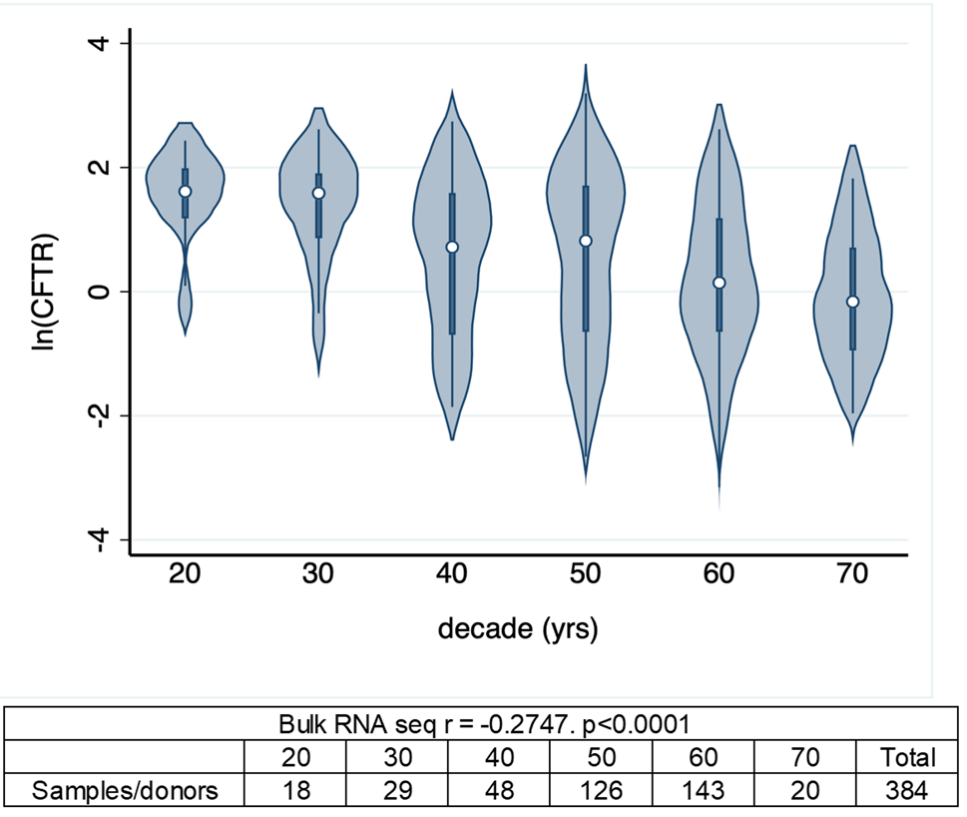
CFTR expression by decade. Bulk RNA sequencing data from lung tissue. Data is from the GTEx portal (18).

**Fig. 2:**
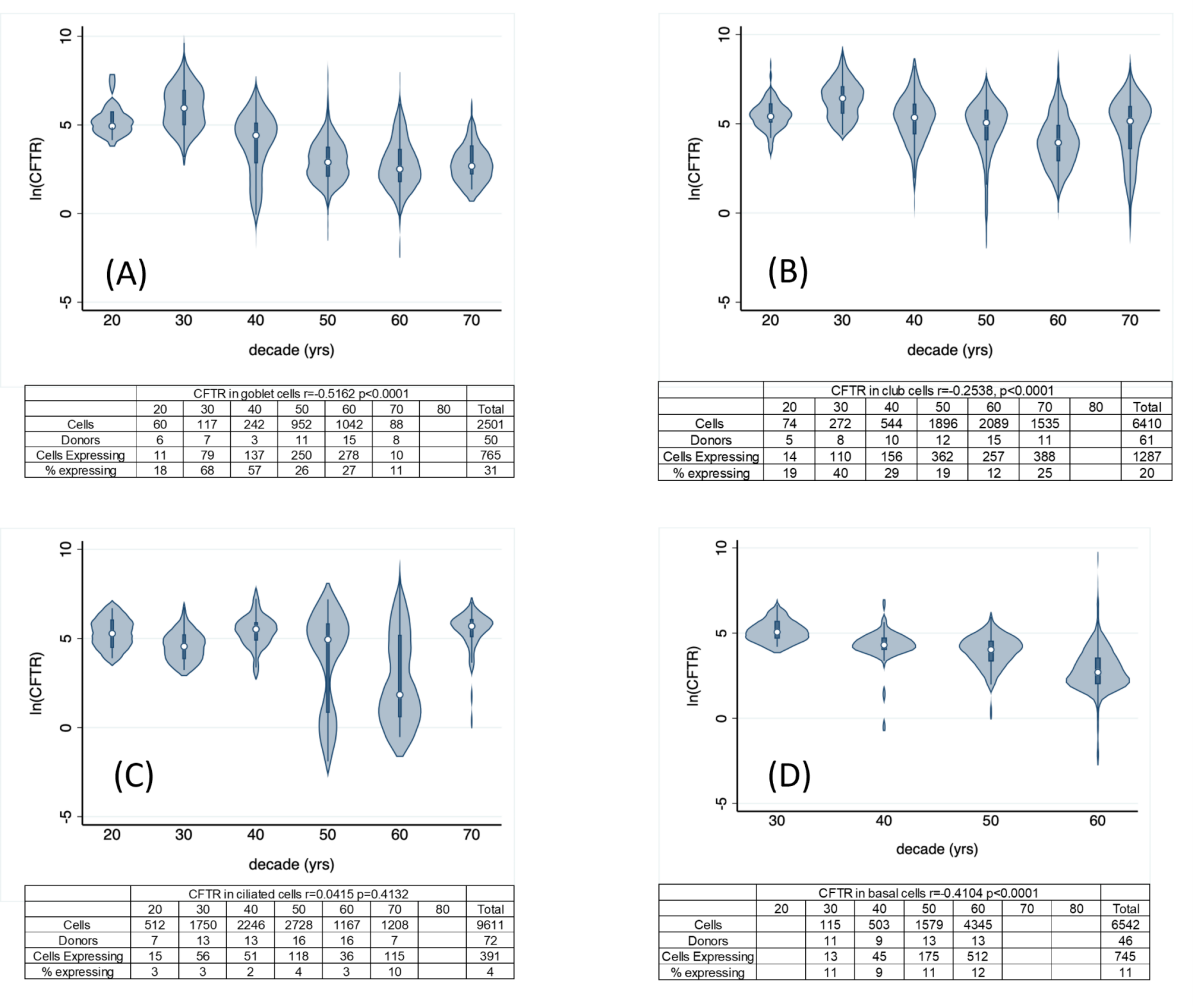
CFTR expression by decade. Single cell seq data from lung tissue in the CELLxGENE single-cell human cell atlas for: (**A**) Goblet cells, (**B**) Club cells, (**C**) Ciliated cells, (**D**) Basal Cells.

CFTR expression decreases with age in the GTEx dataset (*r*= -0.2747, *p* < 0.0001) as shown in Fig. 1. *CFTR* expression also decreases with age in all cell types queried in CELLxGENE, except for ciliated cells (goblet: *r*= -0.5162, *p* < 0.0001; club: *r*= -0.2538, *p* < 0.0001; basal: *r*= -0.4104, *p* = 0.0001), as shown in Fig. 2. Negative r values indicate an inverse relationship (CFTR expression decreases with age). Table 1 shows the correlations based on sex for the cell types included in Fig. 2. The dataset included more female than male donors.

**Table 1:** Correlations between decade age and CFTR expression by sex.

	Male *r*	Female *r*	*n*(M/FM)	Both *r*
Goblet cells	-0.0990	-0.5701***	178/587	-0.5162***
Club cells	-0.3647***	-0.1492***	371/916	-0.2538***
Ciliated cells	-0.2426*	0.1139	95/296	0.0415
Basal cells	0.5481**	-0.3988***	47/696	-0.4104**

(r = Pearson’s r). ***p < 0.0001, **-*p* < 0.001, * -*p* < 0.05.

## Discussion

Our results indicate that *CFTR* expression decreases with age in several key airway cell types including goblet, club, and basal cells. Secretory cells (including goblet and club cells) and basal cells together provide the majority *CFTR* expression in the airways^21,22^. Cl- secretion from CFTR into the ASL establishes the osmotic gradients needed to maintain ASL hydration and functional MC, and therefore decreased *CFTR* expression could mediate the age-related decreases in MC rate noted in previous studies^6–11^. The age-related changes in *CFTR* expression reported here are much larger than those previously described in 24 vs. 3 month-old mice in club, goblet, and ciliated cells (Webtool associated with^17^.)

Our study utilized two publicly available databases that included bulk and single cell sequencing data respectively, along with donor metadata. These databases provide the large numbers and statistical power needed to consider how gene expression varies across the lifespan but provide no route for directly comparing expression to function. A lack of functional correlates makes it difficult to determine whether the changes in expression demonstrated here are physiologically relevant. Previous studies have related the number of cells expressing *CFTR* in bronchial epithelial cell cultures to Cl- secretion, ASL height, ASL absorption rates, and mucociliary transport rate^23,24^. Other limitations to this study include a lack of extensive metadata describing the donors. Lung disease was not characterized in GTEx and thus the sample may contain donors with known or unknown disease. Donors from CELLxGENE were classified as “normal” but the extent of evaluation for disease is not known. Smoking status was not included in our datasets. Our CELLxGENE dataset al.so included a large majority of female donors (see Table 1). The reason for this is not known. *CFTR* represents a druggable target. CFTR potentiator drugs, such as ivacaftor, increase the open probability of the CFTR channel, providing more Cl- secretion from a smaller amount of CFTR. These have been shown to increase MC and decrease obstruction in specific CF genotypes with some retained CFTR expression^20,25^. Other CFTR potentiators have been tested in trials for COPD based on reported associations between smoking and CFTR dysfunction^26^.

Multiple causes may contribute to age-related decreases in MC rate, and other factors likely contribute to the increased proclivity to respiratory infection in older people. Augmentation of MC is an attractive prophylactic option for development since it functions as a host defense against *all inhalable pathogens and particulate toxins*. Identifying the mechanisms contributing most significantly to aging related MC depression in the human lung is a challenging first step to devising such a prophylactic therapy. Identifying aging related trends in the expression of genes important to MC function using large publicly available databases can facilitate the design of more targeted functional studies and provide a more efficient path to a useful therapeutic.

## Materials and methods

### Gene expression analysis

There were 384 samples from the lung where *CFTR* expression and age data were available in the GTEx portal (one measurement per donor). Age data was available in decade increments from 20 to 70 years (v8 metadata). The data described in this manuscript was obtained on approximately 12/12/2022 from the open access portion of the GTex website. (https://gtexportal.org/home/downloads/adult-gtex/bulk_tissue_expression). Expression data was reported in transcripts per million (TPM) which includes normalization. Lung disease was not classified in this dataset. We included only expression data from cells where TPM > 0. All included samples demonstrated *CFTR* expression. We plotted the natural log of expression vs. decade. Correlation was assessed using Pearson’s r - decade vs. natural log of *CFTR* expression. Only donors expressing *CFTR* were included in the correlation. Negative r values indicate inverse correlation. Analysis was performed using Stata/SE 17 (StataCorp, College Station, TX). Correlations and p values were generated using the ‘pwcorr’ command.

We extracted single-cell expression data for club, goblet, ciliated, and basal cells from CELLxGENE database using the publicly available *CuratedAtlasQueryR*^27^ and the *TidySingleCellExperiment*^28^ packages, from donors ages 18–95 in the “normal” (non-diseased) group. Age data was converted to decades from 20 to 80 for analysis. Expression data was normalized as counts per million (CPM). We plotted the natural log of expression vs. decade. The figure includes only expression data from cells where CPM > 0. Data was only included for decade groups with 10 or more cells expressing *CFTR.* Data was downloaded on 2/19/24.

The final analysis datasets are uploaded as supplements.

## Electronic supplementary material

Supplementary Material 1.

Supplementary Material 2.

Supplementary Material 3.

Supplementary Material 4.

Supplementary Material 5.

## Data Availability

The data in this manuscript was obtained from two large, publicly available databases: CellXGene and gTEX. We have provided the specific data reported in supplemental files available with the article.
